# Supplementary figures and images for: A lack of open data standards for large infrastructure projects hampers social-ecological research in the Brazilian Amazon
Source: PeerJ. 2025 Sep 9;13:e19926. doi: 10.7717/peerj.19926 (PMC12428534; doi:10.7717/peerj.19926)

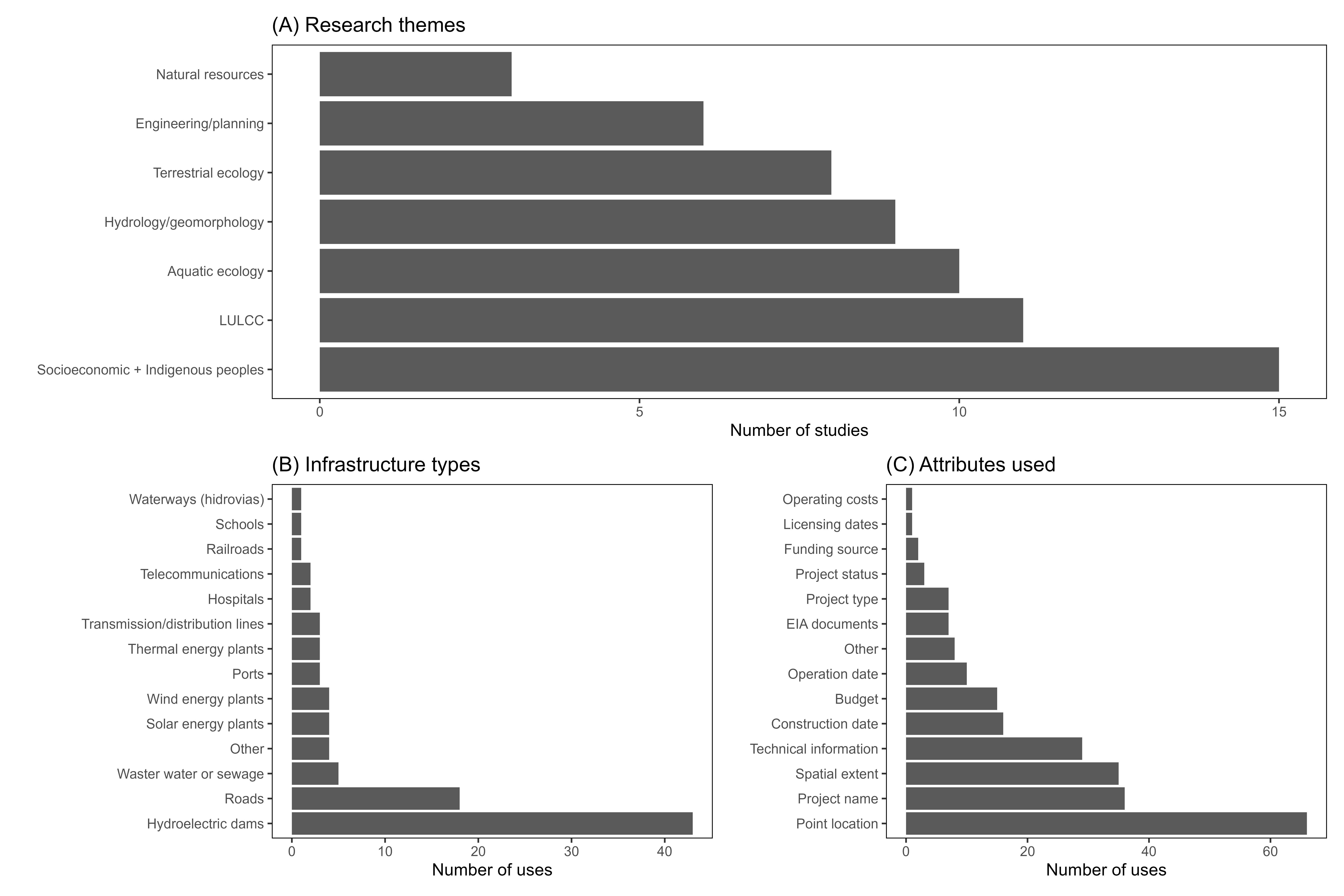

Supplement: Supplemental Information 1 — Number of articles in the systematic literature review conducted in the Web of Science database (WOS) for the 2011-2018 period grouped by (A) thematic research area of the articles (LULC stands for land use/land cover); (B) infrastructure project type researched; and (C) data attributes used in the research (EIA stands for environmental impact assessment). [file peerj-13-19926-s001.png]

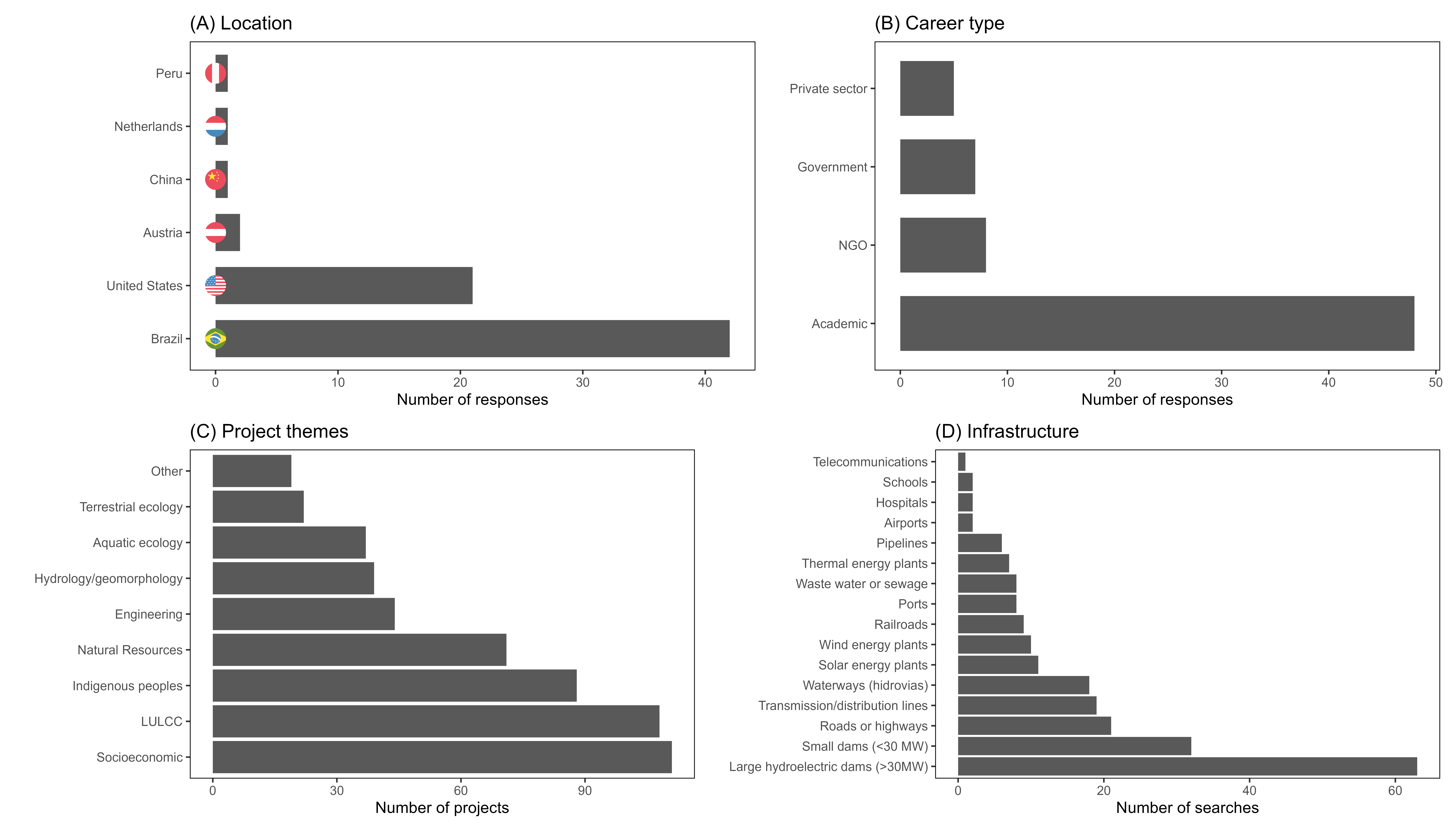

Supplement: Supplemental Information 2 — Survey responses for A) participants’ primary work locations and B) career type; C) themes of the projects for which participants needed infrastructure data. “LULCC” stands for land-use/land-cover change; D) types of infrastructure data searched for. [file peerj-13-19926-s002.png]

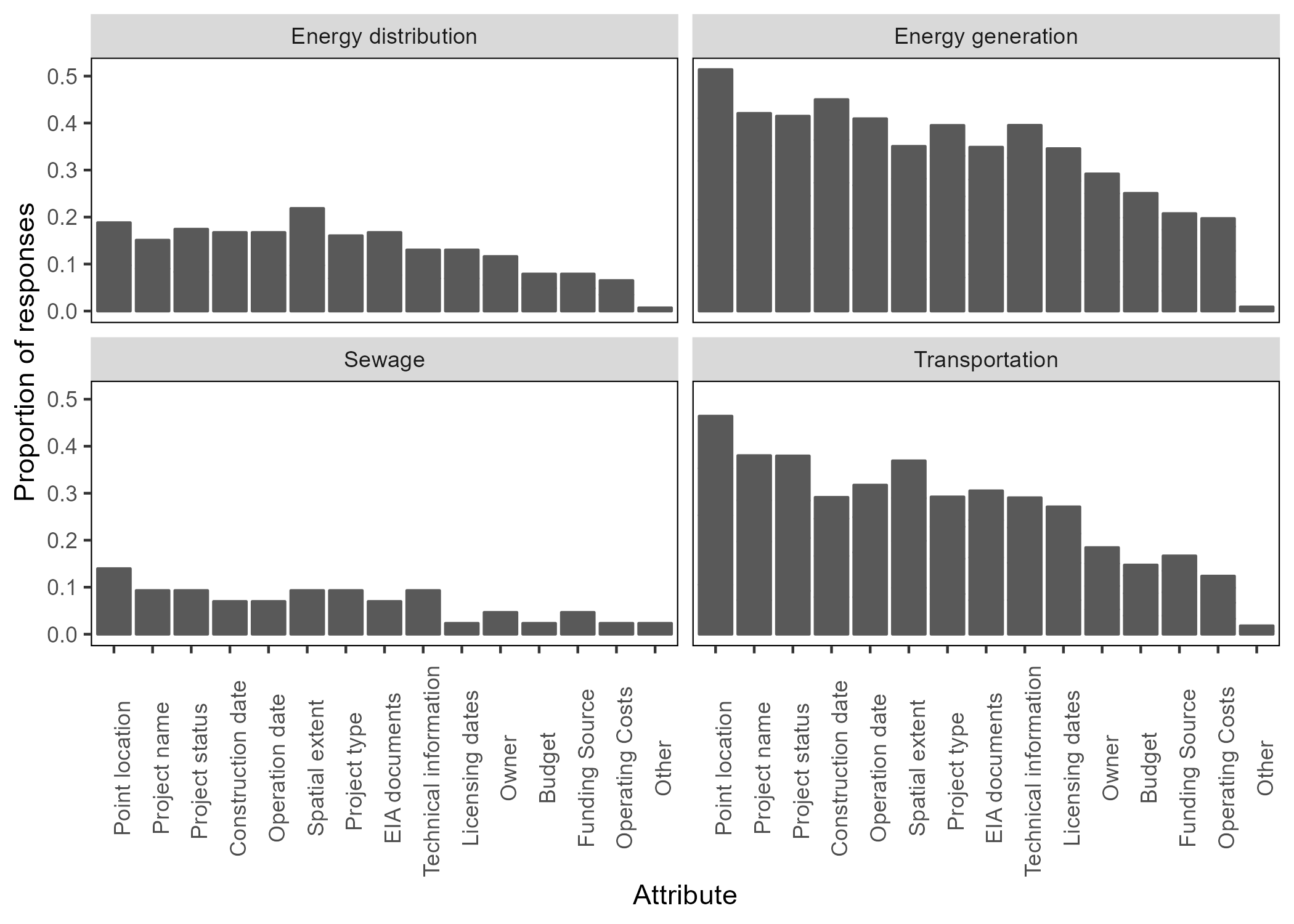

Supplement: Supplemental Information 3 — Projects that had fewer than 10 attribute searches are not shown. Attributes are ordered according to their overall popularity from most to least frequently searched (left to right). Data were grouped into energy distribution (pipelines, transmission/distribution lines), energy generation (large and small dams, solar, thermal, wind), sewage (wastewater and sewage), and transportation (ports, railroads, roads and highways, and waterways/hidrovias). [file peerj-13-19926-s003.png]

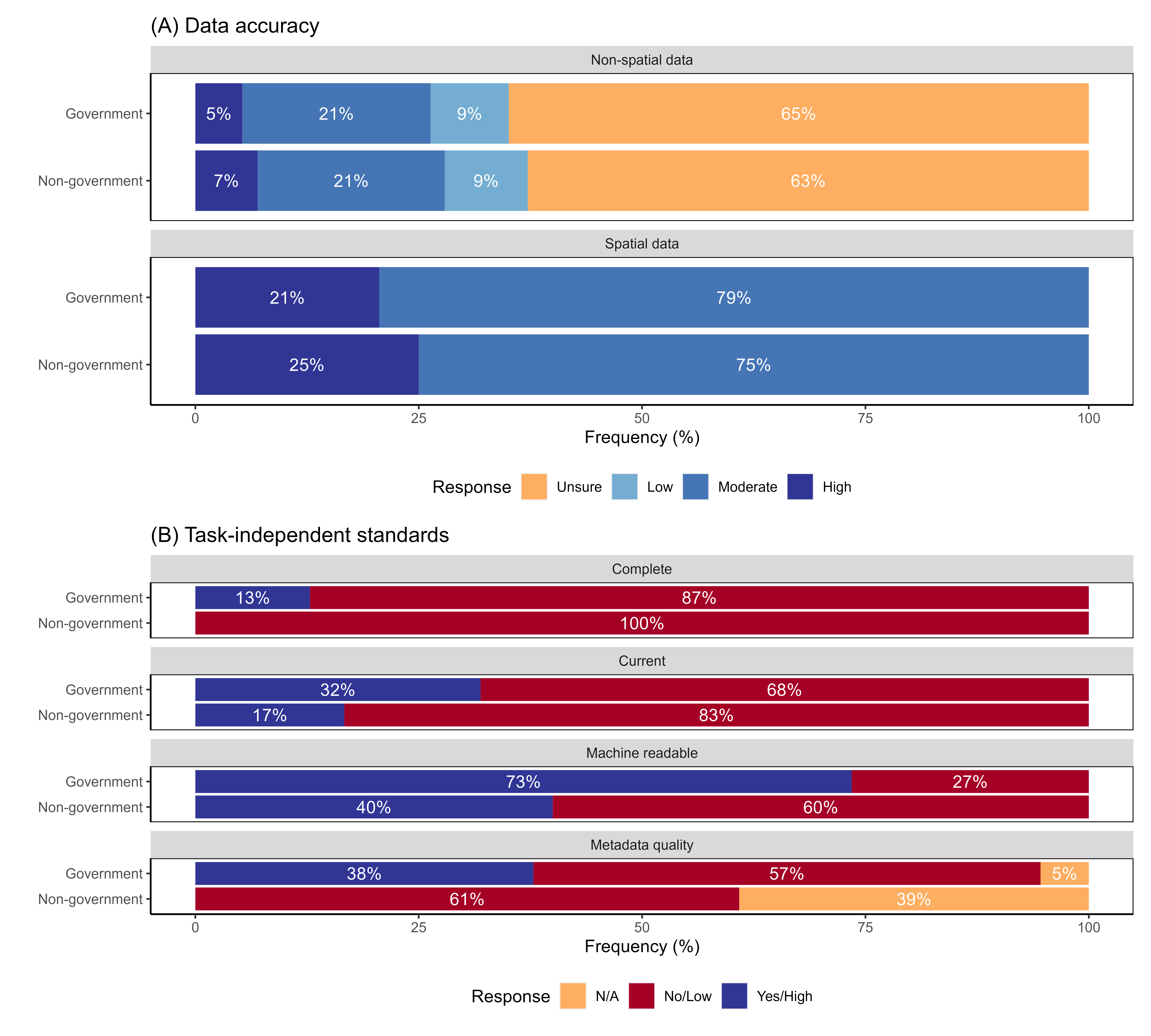

Supplement: Supplemental Information 4 — (A) data accuracy (both spatial and non-spatial components); (B) task-independent standards—completeness, currency (<1 year since update), machine readability, and metadata quality. N/A value for metadata quality indicates respondent did not require metadata for the dataset they used. [file peerj-13-19926-s004.png]

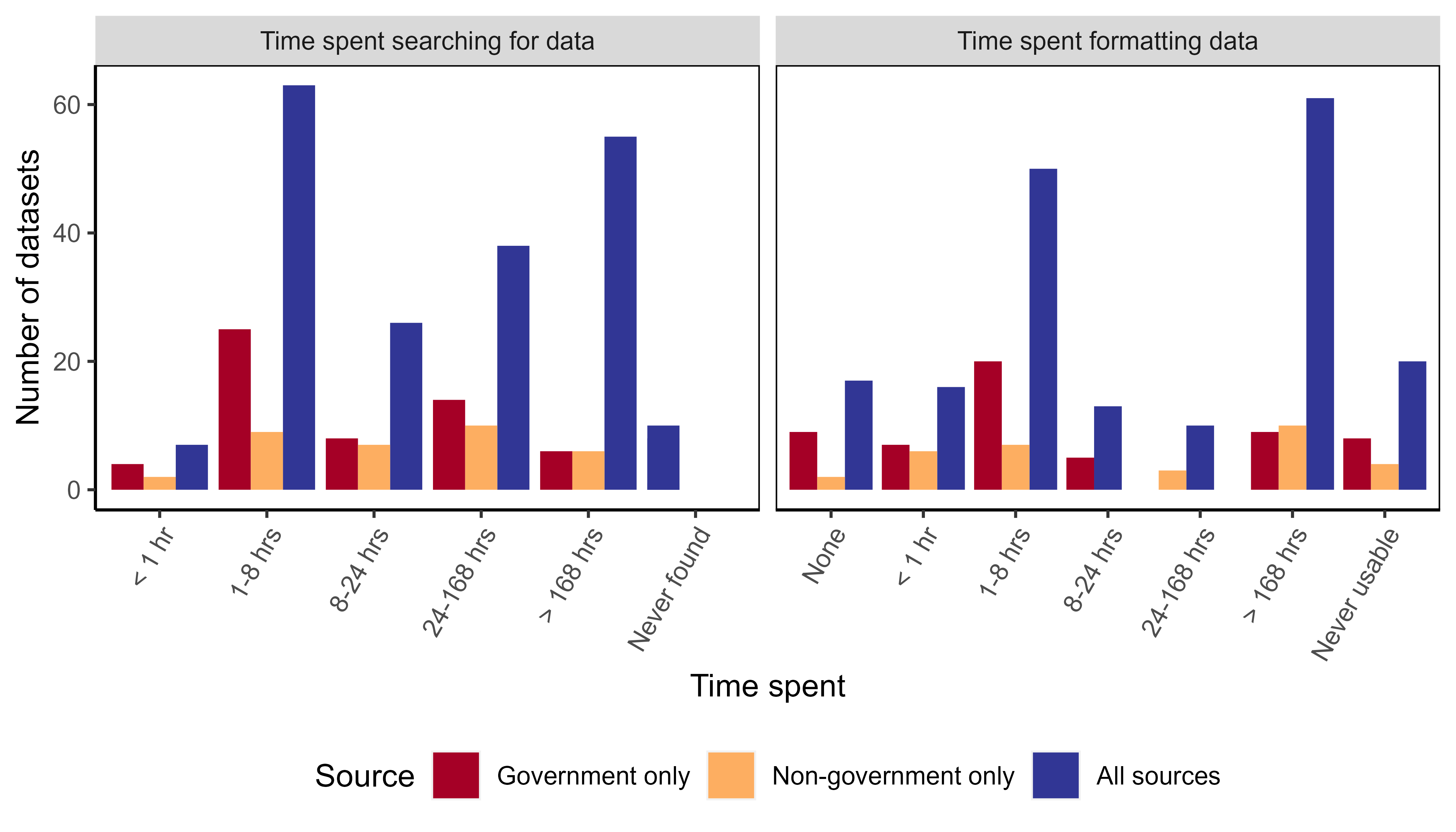

Supplement: Supplemental Information 5 — “All sources” represents the total number of datasets searched for or formatted and includes datasets found only from government or non-government sources and datasets found from both government and non-government sources. As a result, number of data sets from all sources (blue) can exceed the sum of data sets from government only (red) and non-governmental only sources (yellow). [file peerj-13-19926-s005.png]
